# Protective Factors That Maintain Asymptomatic Longevity in Untreated Congenitally Corrected Transposition of Great Arteries

**DOI:** 10.1016/j.cjco.2021.11.004

**Published:** 2021-11-17

**Authors:** Satoshi Masutani, Akari Abe, Yoichi Iwamoto, Hirotaka Ishido

To the Editor:

Congenitally corrected transposition of great arteries (CCTGA) is a rare but important condition, especially in the field of adult congenital heart disease. Although many cases of CCTGA have various associated conditions such as ventricular septal defect (VSD), pulmonary stenosis (PS), and tricuspid valve abnormalities,^1^ there are also cases without such significant associated conditions.^2^ Cases without significant associated conditions have better prognoses, but many of them are still associated with heart failure in patients after 40 to 60 years of age.^1^

We read with interest the report published by Osakada et al., who presented a case report of CCTGA in a patient who was diagnosed at 88 years of age.^2^ This CCTGA case is of the oldest patient reported so far, and a clear contrast-enhanced computed tomography (CT) image was presented. It was reported that this case only had a small atrial septal defect but did not have ventricular septal defect or pulmonary stenosis (PS). From the CT image (Fig. 2F),^2^ however, a right ventricular outflow tract obstruction and supravalvular PS and poststenotic dilation of the pulmonary artery were observed. PS or pulmonary outflow obstruction has been reported as a factor that is associated with better prognosis,^1^^,^^3^ and we are curious as to whether there was pulmonary outflow obstruction or supravalvular PS in this case. The presence or absence of heart murmurs, echocardiographic Doppler findings from the right ventricle to the pulmonary artery, and mitral regurgitation pressure gradient (if it exists) may help bring clarity and deepen the understanding of the protective factors other than situs inversus,^4^ which formed a major factor for CCTGA being undiagnosed without symptoms until 88 years of age.
